# Root biomass fraction of forest-planted sugar maple seedlings varies with climate of population origin across a latitudinal gradient

**DOI:** 10.1007/s00442-026-05978-3

**Published:** 2026-11-10

**Authors:** Rachel C. Putnam, Peter B. Reich

**Affiliations:** 1 University of Minnesota, Minnesota, USA; 2 University of Michigan, Ann Arbor, USA

**Keywords:** Biomass allocation, Allometric, Climate change, Phenotypic plasticity, Range margins

## Abstract

Planting seedlings beyond a species’ or population’s current distribution is a potential management strategy to help plants keep pace with changing climate. The extent to which both climate of origin and spatial climate differences affect seedling traits such as biomass allocation, however, remains poorly understood. Sugar maple (*Acer saccharum*) seedlings from three USA source populations with contrasting climates (from south to north; Missouri, Iowa, Minnesota) were grown for three years at nine forested sites, from Arkansas, USA, to Ontario, Canada, to test hypotheses about ecotypic and biogeographic variation in root biomass fraction (RMF, assessed herein with root biomass/[root biomass + stem biomass]). Sites spanned the north-south transect of the species’ range and extended beyond both northern and southern range margins. Our two main predictions were (i) seedling RMF would increase from southern to northern sites as a reflection of increasing resource constraints, and (ii) for similar reasons, seedlings with origins from further north would on average have both greater RMF and less plasticity in RMF than seedlings from further south. In contrast to our predictions, southern-origin seedlings overall had higher RMF than northern-origin seedlings, with central-origin seedlings intermediate. Southern-origin seedlings had greater RMF at northern than at southern sites, as predicted, but northern and central-origin seedlings did not. Determining how belowground allocation and its plasticity vary across populations and naturally existing biogeographic gradients offers a window into how species may respond to ongoing climate change.

**Supplementary Information:**

The online version contains supplementary material available at https://doi.org/10.1007/s00442-026-05978-3.

## Introduction

Climate change is causing plants in seasonal climates to experience a lengthened growing season as well as novel temperature patterns (Gunderson et al. 2012; Gao et al. 2022). The potential effects of these changes on root biomass allocation in plants, however, is under-studied, particularly in natural environments and across multiple sites (Smithwick et al. 2014; Zhou et al. 2022). Carbon that is allocated to roots has a direct functional impact on plant resource uptake and growth: root structures not only facilitate water and nutrient capture but also store carbon to support future growth. Plants are hypothesized to preferentially allocate biomass towards the acquisition of a limiting resource (Bloom et al. 1985), so the relative proportion of carbon allocated to above- versus belowground tissues can therefore be viewed as a reflection of growth constraints across environmental gradients. Determining the extent to which proportional root biomass allocation shifts across large-scale latitudinal and temperature gradients contributes not only to our ecological knowledge but also has the potential to inform management responses to climate change.

Temperature affects the availability of soil resources such as nutrients and water both directly and indirectly. Soil temperature directly influences rates of root growth, respiration, and nutrient uptake (Pregitzer et al. 2000), while rates of soil nutrient cycling are also temperature-dependent and affect functional nutrient availability (Knoepp and Swank 2002). Temperature and covarying factors such as growing season length also affect soil moisture by influencing rates of evapotranspiration and water demand (Liang et al. 2024). Given the many ways in which temperature affects soil resource availability, we would expect to see clinal variation in root allocation across regional temperature gradients, and indeed, evidence suggests that across broad temperature gradients, roots comprise a higher proportion of total plant biomass in cooler locations (Poorter et al. 2012; Reich et al. 2014; Guo et al. 2021; Zhou et al. 2022).

While shifts in biomass allocation with temperature are apparent from data aggregated across species and biomes, not all species respond the same way. For instance, co-occurring tree species may vary in root traits (Comas and Eissenstat 2009) and in their response to temperature (Lee et al. 2007). In a review of biomass allocation across species and environments, Poorter et al. (2012) concluded that root morphology and architecture (e.g., root branching, specific root length) tends to be more plastic than allocation. Nonetheless, juveniles of temperate species tend to increase allocation to stems and decrease allocation to roots with increasing temperatures (Way and Oren 2010; Poorter et al. 2012; Choury et al. 2025).

Within a species, populations from colder origins also tend to allocate more to roots than populations from warmer origins, even when grown in comparable conditions (Oleksyn et al. 1992; Tenkanen et al. 2021). These phenotypic and genotypic responses to temperature are consistent with the idea that at colder temperatures, greater investment in roots is necessary to acquire more limited nutrient supplies (Zadworny et al. 2016). Other studies, however, have found the opposite pattern (e.g., Montesinos-Navarro et al. 2011; Matías et al. 2014). The lack of consensus and the modest number of relevant studies shows the need for more common garden experiments that span latitudinal gradients to examine intraspecific responses to temperature in the context of interacting environmental factors (De Frenne et al. 2013).

Populations may differ not only in their traits but in the plasticity of those traits (Taeger et al. 2015). Differences between populations in the extent of plasticity could be due in part to evolutionary constraints such as limited gene flow and stressful abiotic conditions reducing plasticity in populations near range margins (Colautti et al. 2010). Between-population differences in plasticity may covary with growth rates (Grassein et al. 2010), and relative growth rate in turn tends to vary with a population’s latitude of origin, though the direction of the relationship is not always consistent from one species to another (e.g., Miyazawa and Lechowicz 2004; Koehler et al. 2012). Data on plasticity of root traits across natural environmental gradients is limited, however, as few studies have explored population differences in root traits of woody species across multiple common gardens.

An additional limitation of prior attempts to describe plasticity in belowground biomass allocation lies in conflating ontogenetically influenced differences in allocation with phenotypic plasticity. Differential rates of growth among plants in experimental treatments may cause substantial size variation among same-aged plants. It has been repeatedly noted that biomass allocation changes with plant size and ontogeny (e.g., Coleman et al. 1994; Reich 2002), and that proportional comparisons of root and shoot biomass between treatments may inflate or misrepresent the treatment effect (McConnaughay and Coleman 1999; Reich 2002). Allometric analyses are therefore the preferred approach for distinguishing between changes in root mass fraction due to experimental conditions (“true plasticity”) and those that are a function of size (Reich 2002; Weiner 2004). Despite this, few studies that measure both above- and below-ground biomass employ an allometric approach.

Sugar maple (*Acer saccharum* Marsh.), a shade-tolerant species common to mesic hardwood forests of the eastern and central United States (Godman et al. 1990), was selected to investigate root allocation across a latitudinal gradient and potential intraspecific variation in response to climate. Sugar maple’s range spans a climate gradient typical of many hardwood species that characterize eastern forests in the United States, with average winter temperatures ranging from − 18 to 10º C, average summer temperatures ranging from 16 to 27º C, and average summer moisture ranging from 380 to 1020 mm (Godman et al. 1990). Genetic variation within the species has been identified for potentially relevant traits such as heat and drought tolerance, onset of dormancy, chilling requirements, timing of leaf flush, and growth rate (Kriebel and Gabriel 1969). Sugar maple has determinant growth and is more strongly influenced by temperature than photoperiod in the timing of bud break and subsequent growth (Olmstead 1951). Furthermore, the structure of sugar maple roots makes the species well-suited for root studies in a natural setting. Sugar maple seedlings are easier to excavate than deep-taprooted species because their roots tend to spread laterally and generally reach a depth of only 20 to 40 cm in the first growing season (Pallardy and Rhoads 1993).

**Fig. 1:**
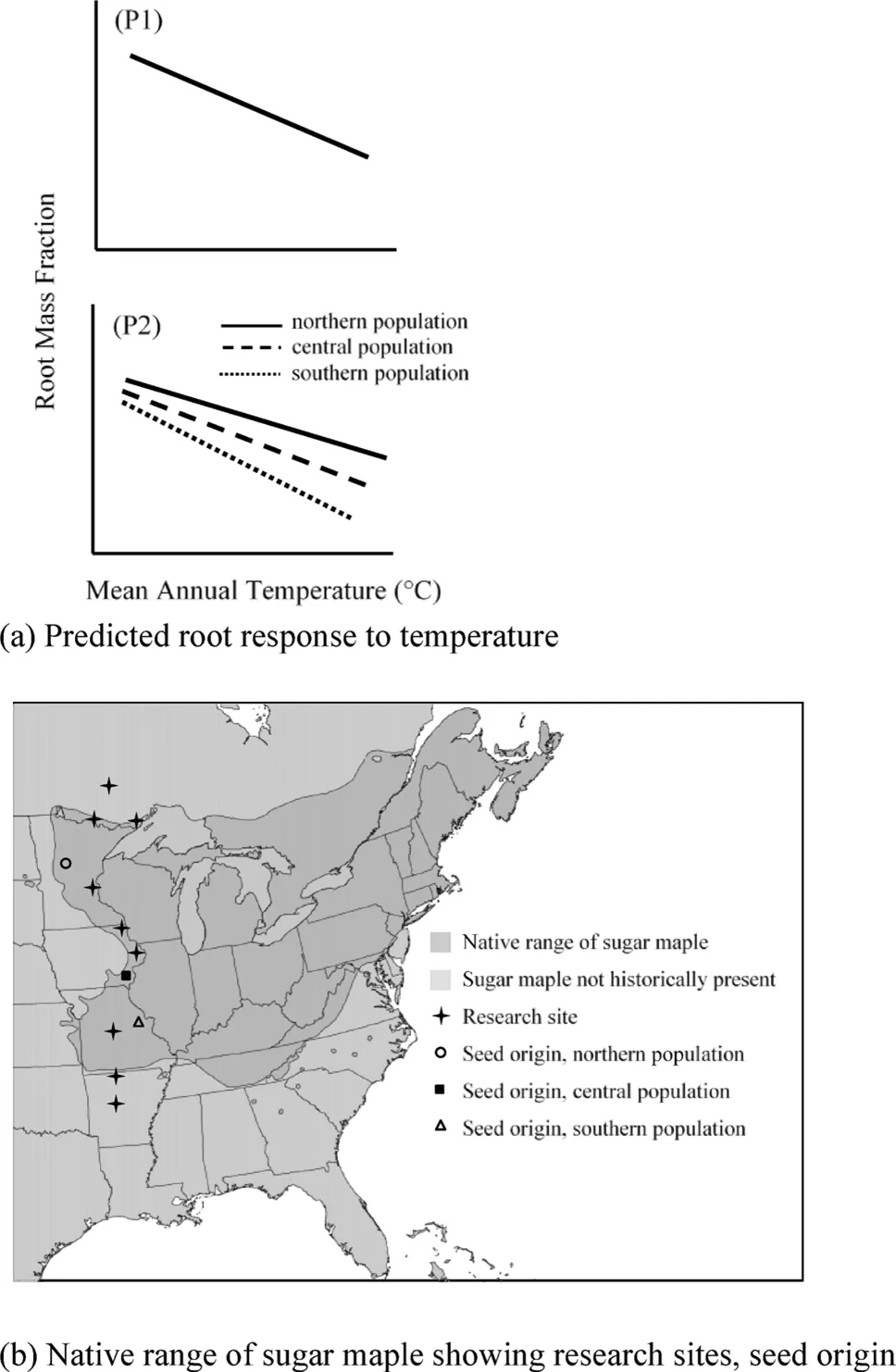
Hypothesized response (**a**) of sugar maple (*Acer saccharum*) seedling root mass fraction to mean annual temperature among all seedlings (P1) and between the three populations (P2) across and beyond the native range of sugar maple (**b**). Regardless of seedling origin, we predicted RMF (root mass/[root mass + stem mass]) would have an inverse relationship with MAT and would be higher at colder sites than warmer ones (P1). We also predicted that after accounting for seedling size, southern-origin seedlings would have a lower proportion of their biomass in roots than central- or northern-origin seedlings across all sites and would exhibit greater plasticity in biomass distribution across sites than central or northern-origin seedlings, as indicated by the steeper slope (P2). To test these predictions, we measured root and shoot biomass of sugar maple seedlings from three populations experimentally planted at nine sites along a north-south transect of the western edge of the species’ range and beyond range margins to the north and south (**b**). Range map is adapted from Little (1971)

We hypothesized sugar maple seedlings would exhibit allocation patterns consistent with local adaption to resource availability and growing conditions in their region of origin. Specifically, we predicted that (P1) RMF would have an inverse relationship with MAT, increasing at colder sites regardless of seedling origin, because roots tend to comprise a greater proportion of total biomass at colder sites (e.g., Reich et al. 2014; Guo et al. 2021). We also predicted (P2) that after accounting for seedling size, southern-origin seedlings would have a lower root biomass (and hence lower RMF) than central- or northern-origin seedlings across all sites (Fig. 1a), because lower allocation to roots relative to stems and leaves is often found in populations originating from warmer regions (Oleksyn et al. 1998; Tenkanen et al. 2021). As part of that prediction, we expect that southern-origin seedlings would exhibit greater plasticity in biomass distribution (Fig. 1a), because plasticity in biomass distribution is generally thought to be higher in populations that occupy more variable environments, are resource-acquisitive, and have higher growth (Grassein et al. 2010).

## Materials and methods

Sugar maple seedlings from three geographically diverse populations were planted at ten sites across a north-to-south transect of sugar maple’s range and beyond range margins (Fig. 1b) as part of an experiment examining the effects of climate and local understory competition on seedling performance and potential constraints on range limits (Putnam and Reich 2017). Young sugar maple were purchased as bare-root one-year-old field-grown seedlings from commercial nurseries in Minnesota, Iowa, and Missouri that used locally-sourced seed. Seedlings from these nurseries (hereafter referred to as the northern, central, and southern populations) originated from Mille Lacs County, Minnesota (5.3º C MAT), Lee County, Iowa (11.1º C MAT), and Lincoln County, Missouri (12.4º C MAT), thus spanning the westernmost extent of sugar maple’s distribution (Fig. 1b) and a 7.1º C range in MAT of origin.

In the spring of 2009, 48 one-meter-radius plots were established at each of ten forested sites. Plots were randomly located either under a closed canopy or in canopy gaps, and every plot had a seedling from each of the three populations. Every site therefore had 144 seedlings initially; a total of 1440 seedlings were planted in all. At the end of the three-year experiment, 714 of these seedlings were still alive. This paper uses data from the nine sites where seedlings from all three populations survived to harvest (ESM Table 1). Of these, five sites spanned a north-south transect of sugar maple’s range on the western edge of the species’ distribution and two each were located beyond the northern and southern range margins, in Ontario and Arkansas, respectively (Fig. 1b).

The latitudinal and climate gradient encompassed by the planting sites is characterized by a 14° C range in mean annual temperature (2.1° to 16.1° C MAT) and 868 mm range in mean total annual precipitation (701 to 1569 mm north to south) (ESM Table 2). Average June/July/August (hereafter referred to as “summer”) temperatures over the past three decades range from 17.5° C in the north to 27.3° C in the south, while cumulative summer precipitation varied from 265 to 303 mm across sites with no clear latitudinal trend (ESM Fig. 1). A site-specific index of moisture availability, which we calculate as summer precipitation/potential evapotranspiration (Thornthwaite 1948), ranges from 0.63 to 0.88 on average, with slightly greater soil moisture availability at higher latitudes (ESM Fig. 1). Conditions in the three years of our experiment generally tracked climate normals closely, though total precipitation (and consequently, soil moisture availability) was notably higher in the central portion of the range than the long-term average (ESM Fig. 1). Temperature and precipitation data for sites in the United States were obtained from the PRISM Climate Group (Oregon State University); the PRISM model, accurate to 2 km, uses weather station data and then interpolates using local data such as elevation (data *available online*)^1^. For the three sites in Ontario, climate data were obtained from the Canadian National Climate Data and Information Archive (Government of Canada) using the weather station nearest each site; accuracy of Canadian weather station data ranges from 15 to 24 km (data *available online*)^2^.

Seedlings were planted in a natural setting within mature forests that had not been recently logged or burned, and were grown in individual fine plastic mesh sleeves to reduce damage from deer browse. Forests at each site were hardwood or hardwood dominated, ranging from oak-hickory forests at southern sites to sugar maple-basswood forests at central sites and birch-aspen-fir forests at northern sites (Table 1). Sugar maple was present in both the canopy and understory at all within-range sites. Other maples that also associate with arbuscular mycorrhiza (*Acer rubrum* and *Acer spicatum*) were present at sites outside the range of sugar maple at varying levels of abundance. Canopy openness was measured with a densiometer; across all sites, closed-canopy plots averaged 7.7% light and canopy gap plots averaged 21% light (Table 1).

**Table 1:** Summary of forest conditions at each site where seedlings were planted, including mean basal area of the forest, canopy composition, mean canopy openness, and average compositional characteristics of the forest understory

	Canopy Characteristics		Characteristics of Forest Understory					
	Canopy Woody Species		Understory Woody Species			Herbaceous Species	Light (% full)	
Site	Dominant Species	Basal area (m^2^/ha)	# Species	Stem count		% Cover	Shade	Gap
				Seedlings	Saplings + trees			
Jessieville, Arkansas Alum Creek Experimental Forest (Alum)	*Pinus echinata* *Quercus alba* *Nyssa sylvatica*	26.4 (+ 5.5)	6.2 (+ 2.2)	94.4 (+ 46.5)	1.5 (+ 2.2)	2.8 (+ 8.5)	9.2 (+ 2.2)	17.2 (+ 4.1)
Jasper, Arkansas Henry R. Koen Experimental Forest (Koen)	*Quercus alba* *Quercus velutina* *Nyssa sylvatica*	20.2 (+ 6.5)	11.9 (+ 3.0)	64.2 (+ 30.6)	2.6 (+ 3.3)	6.4 (+ 7.7)	9.7 (+ 3.3)	23.2 (+ 6.7)
Ashland, Missouri Baskett Research & Education Area (Brea)	*Quercus alba* *Quercus rubra* *Carya ovata*	22.6 (+ 5.9)	10.3 (+ 3.9)	84.4 (+ 88.4)	1.8 (+ 3.2)	21.8 (+ 19.3)	5.3 (+ 1.9)	12.8 (+ 4.6)
Muscatine, Iowa Wildcat Den State Park (Wild)	*Quercus alba* *Acer saccharum* *Juglans nigra*	22.6 (+ 8.4)	6.5 (+ 3.1)	43.5 (+ 47.3)	0.3 (+ 0.6)	62.9 (+ 35.6)	6.8 (+ 2.6)	22.2 (+ 9.8)
New Albin, Iowa Fish Farm Mounds State Preserve (Fish)	*Quercus rubra* *Ulmus rubra* *Quercus alba*	26.3 (+ 8.2)	8.3 (+ 3.0)	88.0 (+ 67.4)	2.4 (+ 3.3)	77.8 (+ 39.6)	5.4 (+ 2.4)	11.5 (+ 5.8)
Marine-on-St. Croix, MN Pine Needles tract (Pine)	*Pinus strobus* *Acer saccharum* *Tilia americana*	31.4 (+ 9.6)	8.4 (+ 3.6)	68.2 (+ 77.0)	1.3 (+ 3.1)	39.9 (+ 30.6)	6.3 (+ 2.1)	20.2 (+ 11.9)
Neebing, Ontario Private land (Tbay)	*Acer saccharum* *Populus tremuloides* *Abies balsamea*	25.5 (+ 9.0)	5.7 (+ 2.6)	174.7 (+ 122.2)	10.6 (+ 8.6)	18.8 (+ 23.0)	5.9 (+ 3.0)	18.0 (+ 9.9)
Fort Frances, Ontario Crown land (Fort)	*Populus tremuloides* *Abies balsamea* *Betula papyrifera*	23.3 (+ 8.5)	8.6 (+ 2.0)	79.4 (+ 29.6)	23.6 (+ 16.2)	53.5 (+ 31.5)	9.2 (+ 3.7)	30.2 (+ 14.9)
Dryden, Ontario Crown land (Dryd)	*Fraxinus nigra* *Betula papyrifera* *Abies balsamea*	23.5 (+ 10.6)	5.1 (+ 1.6)	30.3 (+ 20.0)	21.8 (+ 11.7)	91.4 (+ 45.0)	11.1 (+ 7.0)	36.1 (+ 18.3)

*Notes*: Tree density surrounding each plot was measured using a 10-factor wedge prism to estimate basal area of canopy trees (m^2^/ha). Understory woody species were counted within a 2-m radius of plot center, and density was estimated using stem counts, here summarized into seedlings (those below 1.5 m in height) and saplings and trees (those above 1.5 m in height). Percent cover of herbaceous species within a 1-m radius of plot center was visually estimated for each species (totals could exceed 100%). Average percent light penetrating the canopy at each site was calculated by averaging plot-level densitometer readings across years for each canopy cover type: closed canopy (“shade”) and open canopy (“gap”)

At the time of planting, roots of the sugar maple seedlings were trimmed to 25 cm so that all seedlings initially had roots at the same depth; most seedlings had roots that required trimming. This is common practice in planting, especially when roots are larger (Stimm et al. 2013), as was the case here. Although root trimming may have had an effect on relative biomass allocation in the short term, the three-year experiment provided a relatively long time for each seedling to re-establish biomass allocation patterns that reflect local conditions and each population’s genetic predisposition.

After three complete growing seasons, all remaining living seedlings were dug up by hand in the spring of 2012 while they were still dormant and before aboveground growth commenced. For this study, therefore, “total seedling mass” refers to the combined mass of non-photosynthetic shoots and roots, and we define root mass fraction (RMF) as the dry mass of roots divided by the combined biomass of roots and aboveground stems. The majority of roots (both fine and coarse roots) were successfully unearthed. Seedling roots were cleaned immediately post-harvest when necessary to remove soil, then kiln dried at 75° C for 48 h. Dry mass measurements were recorded for shoots (stems only; leaves were not present) and combined fine and coarse roots.

The large distances between sites and interest in having three full field seasons with which to assess growth and survival prevented us from gathering data on biomass allocation to leaves as well. Nonetheless, prior research with juvenile sugar maple and other northern hardwoods in central North America has shown different allocation to stems versus roots among species and among conditions, both with naturally occurring seedlings in the field and in controlled experiments (Walters et al. 1993; Walters and Reich 1997, 2000; Reich et al. 1998). This suggests that the relative distribution of biomass in stems versus roots in our present multi-site and multi-provenance study should be informative and is likely reflective of a genetic predisposition (i.e., differences among provenances) and/or a plastic response (i.e., differences among sites) that likely involves the relative value of seeking belowground versus aboveground resources.

Initial seedling size differed among populations: on average, seedling height and root collar measurements were greatest for southern population seedlings, intermediate for northern population seedlings, and smallest for central population seedlings. Within-population variation in the size of the seedlings, all same-aged, enabled an allometric analysis of how the distribution of biomass to roots shifts with seedling size. This experiment does not address changes in allometry over time, however, as all seedlings were collected in a single harvest.

Proportional biomass distribution to roots versus shoots was analyzed using an allometric approach that accounts for potential variation in biomass distribution with plant size. Log-transformed dry mass of roots was the response variable and log-transformed dry mass of non-photosynthetic shoots was included as a predictor in all models. Analyses employed a standard least squares mixed model (REML) approach; both site and plot (nested within site) were entered as random effects to account for site-specific variation in seedling performance unrelated to the climate gradient.

We used AIC and BIC values to compare the models using three potential climate variables: mean summer temperature, mean summer moisture availability, and mean summer precipitation. All three models had similar AIC and BIC values, but of these, the model with mean summer temperature (AIC − 664.5, BIC − 488.5) was marginally superior to the other two models (AIC − 662.3 and − 661.3, BIC − 486.3 and − 485.8). Because our distribution of research sites was explicitly designed to encompass a temperature gradient and address hypotheses on temperature effects on root allocation, we therefore focused our analyses on temperature. We used mean summer temperature rather than mean annual temperature, as the former reflects conditions during the growing season when the majority of tissue allocation occurs.

We then compared the full model, with continuous variables temperature and log shoot mass and categorical variables population, clip treatment, and canopy cover type, as well as all two- and three-way interactions (ESM Table 3), to a simplified model without clip treatment or the canopy cover type (Table 2). While the canopy cover type did have a significant effect on seedling root mass (ESM Table 3), differences between gaps and closed canopy plots in root mass fraction were tiny (both averaged 0.66), there were no significant interactions with temperature, and the AIC and BIC values were less negative for this model (-664.5, -488.5) than those of the more conservative model where clip treatment and canopy cover type were omitted (-687.4, -620.2). Hence we selected the simpler model to be shown as a table in the main text. Data were analyzed with JMP Student Edition 19.0.1 statistical software (SAS Institute Inc.).

## Results

**Fig. 2:**
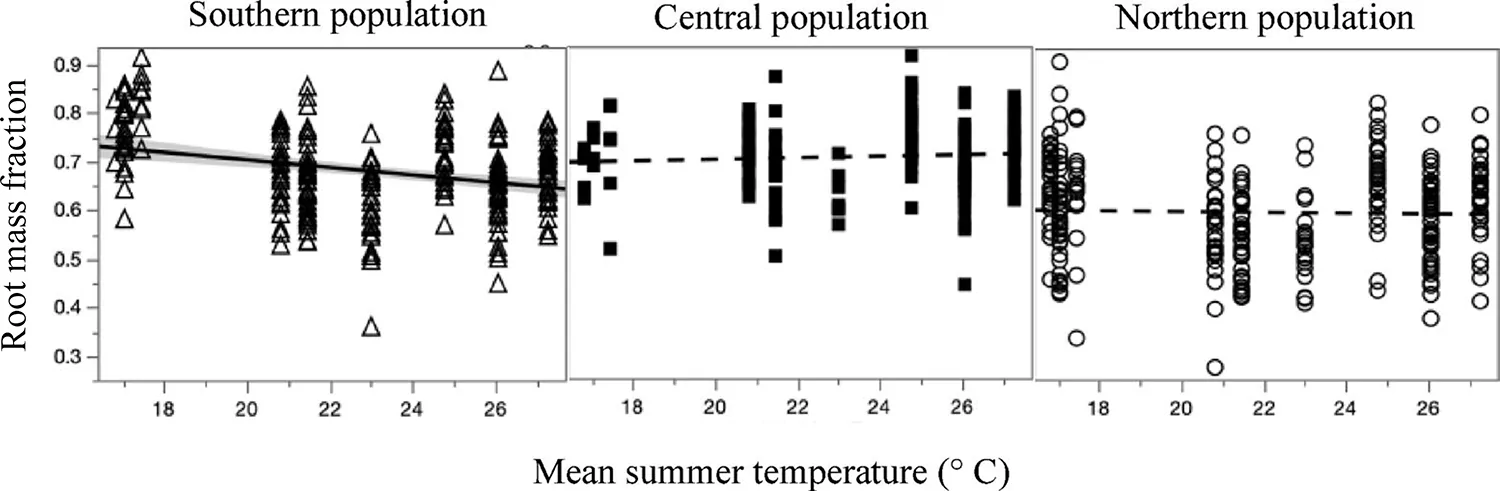
The three populations differed in the degree to which their mean root mass fraction (RMF, (root mass/[root mass + stem mass])) varied across the nine sites, evidence of a significant interaction between populations and the temperature of planting sites (see Table 2). While RMF fluctuated from site to site, there was statistically significant inverse relationship between average summer temperature and RMF for the southern population seedlings, while northern and central populations showed no significant trend with RMF in response to temperature. Each panel has identical axes to facilitate comparison across sites

Population origin affected patterns of root biomass distribution, both as a main effect and through interactive effects with temperature at planting sites (Table 2). The populations differed significantly in root mass (*F*_2,575_= 110, *p* < 0.001), but in contrast to our hypothesis, seedlings from the southern population consistently had higher root mass at a given shoot mass than similarly-sized northern population seedlings (Fig. 2). The central population, meanwhile, was intermediate: based on the model in Table 2, at a standardized shoot mass and summer temperature, it had greater root mass (log_10_ root mass in g = 0.59) than the northern population (0.49) but less than the southern population (0.73). These differences were all significant at *p* = 0.0001 using Tukey’s posthoc test.

**Table 2:** The effects of population (northern, central, and southern origin) and temperature (average summer temperature over the three years of the study at each transplant site) on log-transformed root mass of sugar maple seedlings, accounting for differences in shoot mass (*R*^*2*^ = 0.912, *N* = 683). Seedlings were grown at nine forested sites spanning a latitudinal gradient with a 14 °C range in MAT. In this mixed-effects model, both site and plot (nested within site) were entered as random effects to account for site-specific variation in seedling performance unrelated to temperature, and the random effects for plot were significant (*p* < 0.01). Even after taking plot-level effects and differences in seedling shoot mass into account, though, there were still significant interactive effects of population and temperature on seedling root mass

Fixed effect	df	F	*p*	
Population	2, 575	109.6	**< 0.001**	
Shoot mass	1, 671	1127.1	**< 0.001**	
Temperature	1, 8	0.6	0.464	
Population x Shoot mass	2, 588	3.7	**0.025**	
Population x Temperature	2, 570	4.6	**0.010**	
Shoot mass x Temperature	1, 670	1.0	0.307	
Population x Shoot mass x Temperature	2, 618	2.5	0.085	

**Fig. 3:**
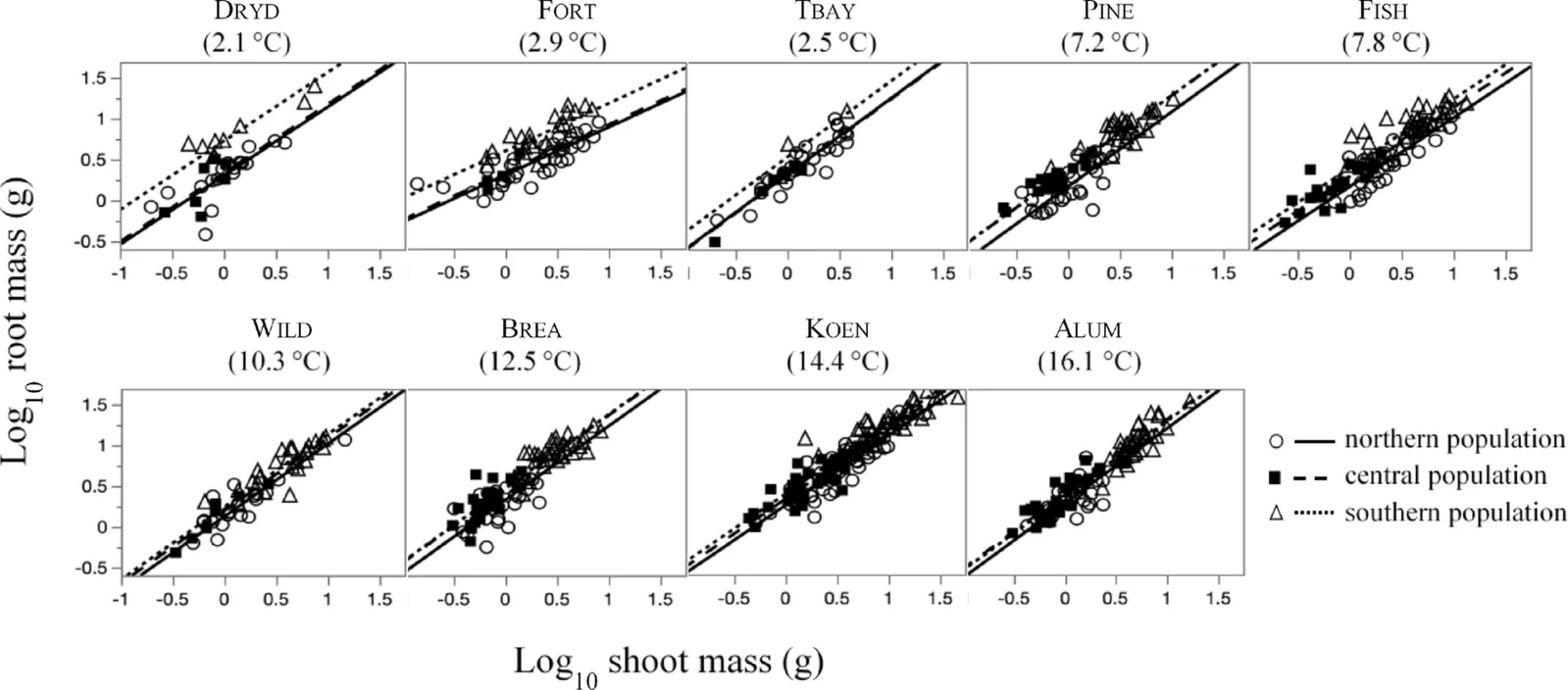
Across sites spanning a latitudinal and temperature gradient (north to south, left to right), the difference in the allometric relationship of root mass to shoot mass (log transformed) between populations increased as MAT decreased. At every site, seedlings from the southern population tended to have higher root mass at a given shoot mass than northern or central population seedlings, but this difference was greatest at the northernmost sites. Because slopes of the three populations did not differ at any site, site-specific slopes of the pooled populations were used at each site. Each panel has identical axes to facilitate comparison across sites

The difference between populations in allometric patterns of root mass distribution was greatest at northern sites, where southern population seedlings had significantly more root mass than similarly-sized seedlings from the northern population, while central population seedlings had root mass that was intermediate between the others. At the southernmost site, in contrast, northern, central, and southern population seedlings with similar shoot mass had approximately equal root mass (population $\:\times\:$ temperature interaction, *F*_2,670_ = 4.6, *p* = 0.010, Table 2; Figs. 2 and 3). This underscores the importance of looking at biomass distribution patterns at the population level, as populations’ significantly different responses to temperature may be obscured at the species level. Significant differences between populations in the allometric trajectory of how root mass changed with size (population $\:\times\:$ shoot mass interaction, *F*_2,588_ = 3.7, *p* = 0.025) reflect a different allocation strategy in the southern population, which was “rootier” in general than the northern population but especially at smaller seedling sizes (Fig. 2). Additionally, the southern population showed shifts in average RMF with the temperature gradients (Fig. 2), but the central and northern populations did not.

## Discussion

Root biomass fraction was expected to shift spatially with latitude and temperature, since growing conditions, growing season length, and the degree to which certain resources are limiting often change across latitudinal and temperature gradients. Multi-species analyses indicate that at the community level, root biomass allocation in woody species tends to be greater at lower temperatures across broad regional to global scales (Poorter et al. 2012; Reich et al. 2014; Guo et al. 2021). Less data is available on species-specific patterns in the root biomass allocation of woody seedlings in response to temperature, yet this is a critical stage to understand as plants respond to changing climate. We found no evidence that temperature across a latitudinal gradient consistently affects phenotypic variation in root biomass fraction of sugar maple seedlings. Instead, we found intraspecific variation in the degree to which biomass distribution patterns shifted with temperature: only the southern population responded in a manner consistent with global patterns, showing greater proportional root biomass at cold northern sites.

We predicted that across the latitudinal gradient, seedlings from southern populations would exhibit consistently lower root biomass than those from northern populations, mirroring global biogeographic patterns of root biomass allocation. In contrast to this expectation, however, we found that regardless of seedling size, southern seedlings had higher root mass than northern seedlings across all sites. Furthermore, seedlings from the central population had intermediate patterns of proportional root mass. The few other studies that have measured within-species variation in proportional root biomass for woody seedlings have found conflicting trends: for example, Matías et al. (2014) found higher proportional root mass in provenances of *Pinus sylvestris* seedlings from warmer regions, while Tenkanen et al. (2021) found the opposite pattern in *Betula pendula* seedlings.

Potentially adaptive differences in root biomass between populations such as those we observed could arise from regional differences in soil resources. Plants tend to increase the proportion of roots relative to aboveground biomass under less fertile conditions (Reich 2002; Kobe et al. 2010), and within a species, populations originating from sites with low-nutrient soils may have a higher proportion of biomass in roots in both nutrient-rich and nutrient-poor soils than populations from sites where nutrients are not limiting (Burgess et al. 2015). We did not directly measure soil nutrients in this study, so we cannot assess the degree to which soil fertility varies from north to south at the western edge of sugar maple’s range. It is possible that seedlings experienced different initial growing conditions in their nurseries of origin that affected their biomass allocation to roots, but seedlings did have three full growing seasons at experimental sites in which they were not watered or fertilized. Seedlings, including sugar maple, are capable of relatively rapid adjustments to root versus shoot versus leaf biomass allocation (Walters et al. 1993; Sendall et al. 2015), and it is likely that seedlings had the opportunity to re-establish root: shoot relationships that reflect in situ growing conditions and potential genetic differentiation.

Soil moisture is another resource that may vary geographically, though one study suggested there is little evidence of shifts in biomass distribution in response to water availability after accounting for plant size (Reich 2002). In periodically dry environments, water limitation may be instead be reflected by plasticity in root morphology (Padilla et al. 2013). A few studies have found genetic variation in biomass allocation patterns that match soil moisture differences in the populations’ regions of origin, with higher proportion of roots in genotypes from regions with lower soil moisture (Richter et al. 2012; Kuster et al. 2013). While there is little geographic pattern in average annual moisture availability among populations’ regions of origin, there are seasonal and inter-annual differences in soil moisture experienced by the three source populations). For sugar maple, the higher root mass of southern population seedlings may allow this drought-sensitive species to survive periodic low soil moisture, which occurs more frequently at the southern edge of its range (ESM Table 4).

Variation in soil chemistry and biology, especially involving the presence of arbuscular mycorrhizae, have also been shown to influence sugar maple growth and survival (Carteron et al. 2020; Chamard et al. 2024). Hence, geographic differences in these properties in our study might have also influenced the distribution of biomass in roots and shoots, especially if that distribution was related to resource supply. If lack of arbuscular mycorrhizae (AM) fungi constrained soil resource uptake, one might posit that there should be greater relative allocation towards roots outside the sugar maple range (where likelihood of infection by AM fungi would often be much lower due to greater dominance by tree species that partner with ectomycorrhizal fungi and very low abundance of other *Acer* genera). However, there was no tendency for RMF to differ within the range of sugar maple versus beyond (*p* > 0.10). Moreover, two populations showed no geographic pattern in RMF and the one that did (the southern population) tended towards higher RMF beyond the range to the north and lower RMF beyond the range to the south, suggesting that AM fungi availability was likely not a major factor in our study.

Genetic variation among plant populations may lead not only to differing patterns of biomass distribution but also differences in the degree of plasticity they express in response to temperature and resource gradients. The southern sugar maple population, whose seedlings had higher root biomass at northern than southern sites, showed greater plasticity in root biomass than seedlings from the other populations, whose proportional root biomass stayed relatively constant across sites. Higher plasticity of roots in the southern population, which had a higher relative growth rate potential than the northern population (Putnam 2015), is consistent with other studies that have found higher intraspecific trait plasticity in populations with rapid resource capture and growth strategies (e.g., Reinhardt et al. 2011; Solé-Medina et al. 2022). Very few studies examining plasticity in biomass distribution have used an allometric approach, however (but see Troughton 1961; Ledig et al. 1970), which is critical in differentiating true plasticity from shifts in biomass that are a function of size rather than environment per se (Reich 2002; Weiner 2004). This study, which took a quasi-allometric approach and controlled for size when comparing seedling root biomass, supports the conclusion that differences among populations in biomass distribution to roots and the plasticity of those responses go beyond size-dependent effects.

Experiments across latitudinal gradients offer important insights into the effects of temperature (De Frenne et al. 2013), but interpreting results from such experiments is also implicitly challenging, as these gradients encompass multiple co-varying environmental factors such as soil moisture availability and photoperiod. Furthermore, the temperature and moisture gradients reflected by the climate data almost certainly do not reflect the microclimate experienced by the seedlings at each site. Nevertheless, these coarse climate measurements are a useful stand-in for the differences seedlings experienced across regional temperature and moisture gradients. In our study, seedlings of the southern population showed significant shifts in RMF in relation to summer temperature, but not the three-year average of soil moisture availability. Therefore, it seems likely that in this case, the plasticity in proportional root biomass distribution expressed by southern population seedlings at northern sites was in response to temperature rather than a water availability gradient.

One potential way that adaptive differences in response to temperature might be expressed is through variation in patterns of biomass allocation among populations, but prior to our experiment there has been very little experimental evidence of this. One study found population differences in the responsiveness of root length to a greenhouse warming treatment (Matías et al. 2014), but other studies that looked for shifts in root biomass of provenances using experimentally manipulated temperature treatments failed to find a significant interaction between temperature and latitude of population origin (Richter et al. 2012; Kuster et al. 2013). None of these studies, however, used an allometric analysis to distinguish between the effects of size differences (‘apparent plasticity’, McConnaughay and Coleman 1999) and true plasticity. Without accounting for differences in seedling size, population differences across treatments do not constitute evidence of true plasticity (Reich 2002; Weiner 2004).

Because so few studies have taken an allometric approach in examining plasticity in root biomass, it is difficult to determine the generality of our results. It may be that plasticity of biomass distribution among populations in response to temperature is uncommon if there is an easier or less costly way for plants to shift resource acquisition (such as by shifting the morphology of leaves or roots, e.g., specific leaf area or specific root length, or increasing the nutrient uptake potential of roots by allocating to cellular nutrient uptake mechanisms) than at the coarse level of above- and belowground biomass distribution (Reich 2002). Alternatively, plasticity of biomass distribution in response to temperature gradients may happen only when plants are exposed to a broader temperature gradient than is usually experimentally tested. Further study is needed to better understand whether and how plasticity of biomass distribution operates across large-scale temperature gradients.

The proportional root biomass response of individual species to temperature gradients has been inconsistent among studies, in contrast to the general global pattern of greater root biomass at colder sites. Several studies using experimentally or passively warmed outdoor chambers (raising temperatures 2 to 5 °C above ambient) failed to find a direct effect of temperature on roots or on proportional biomass distribution (Richter et al. 2012; Kuster et al. 2013; Taeger et al. 2015), in line with the results from our large-scale common garden study. Those studies that found a significant effect of temperature on roots are conflicting: one found a positive effect (Matías et al. 2014) while another detected a negative effect of warmer temperature on roots (Way and Sage 2008). It seems, therefore, that it is premature to use the broad regional relationship between root biomass and temperature as a predictor of root biomass dynamics at the species level.

There are several possible explanations for why plant species seem to differ in the direction and degree to which they change biomass allocation to roots across temperature gradients. Broad global patterns often emerge only across very large gradients; the synthesis of Reich et al. (2014), for instance, spans over 40 °C MAT. Across a narrower range of temperature, species-level relationships between biomass and temperature could be opposite that of the overall trend yet still fit within the range of variation encompassed by the global relationship between temperature and biomass allocation. Biomass allocation could also be relatively stable at the species level yet still contribute to a global pattern of increasing root biomass allocation at colder temperatures if more northerly species tended to have higher biomass allocation to roots than more southerly species. One study found root: shoot ratios of tundra species remained stable along an elevation gradient, but noted that in contrast to global biomass allocation analyses, community composition was very similar across sites (Blume-Werry et al. 2018). Finally, species patterns also do not capture the complexity of intraspecific variation among populations, which could have opposing patterns of biomass distribution responses to temperature. Global patterns may therefore be driven by interspecific, rather than intraspecific, differences in response to broad temperature gradients.

## Conclusion

The effects of climate change on plants may be expressed through phenotypic plasticity in traits as well as genetic differences in traits (Franks et al. 2014), and determining the plasticity of belowground allocation across naturally existing resource gradients therefore offers one window into how species may respond to ongoing climate change (e.g., McConnaughay and Coleman 1999; Reich 2002; Poorter et al. 2012). Though differences in biomass allocation across environmental gradients have been noted both between species (e.g., Portsmuth and Niinemets 2007; Grassein et al. 2010) and within species (e.g., Montesinos-Navarro et al. 2011; Matías et al. 2014), it is important to account for differences in plant size to accurately detect evidence of allocation plasticity (Weiner 2004). This study found differences among populations related to their climate origin, but found shifts in biomass allocation across environments within only one population, suggesting that there may be costs to adjusting biomass allocation to roots that in many cases exceed the benefits of such plasticity. Instead, a plastic response in biomass allocation may be selected for only in populations occupying highly variable or resource-rich environments. Conflicting results from this and the handful of other studies to gather data on both genotypic differences and their plasticity vis-à-vis root biomass allocation across natural environmental gradients point to the need for additional research on allocation to roots within and among species along resource and climate gradients in order to arrive at a more comprehensive understanding of this fundamental issue.

## Supplementary Information

Below is the link to the electronic supplementary material.

Supplementary Material 1

## Data Availability

The dataset from this study is available from the corresponding author on reasonable request.

## Footnotes

1
http://prism.oregonstate.edu.

2
http://climate.weather.gc.ca.
