# Supplementary material for: Root biomass fraction of forest-planted sugar maple seedlings varies with climate of population origin across a latitudinal gradient
Source: Oecologia. 2026 Nov 10;208(12):166. doi: 10.1007/s00442-026-05978-3 (PMC13751729; doi:10.1007/s00442-026-05978-3)
Supplement: Supplementary file 1 [file 442_2026_5978_MOESM1_ESM.docx]

(c)

Mean Summer Precipitation (mm)

Latitude

(d)

Mean Summer Avail. Soil Moisture

Latitude

b)

Mean Winter Min. Temperature (° C)

Latitude

Mean Summer Temperature (° C)

Latitude

(a)

**ESM Figure 1** Comparisons of weather data averaged over the three years of the experiment (dashed line) and 30-year climate normals (solid line) for average summer (June/July/August) temperature (a), average winter (December/January/February) minimum temperature (b), total summer precipitation (c), and summer available soil moisture (d) at each of the sites. Climate data obtained from the PRISM Climate Group (for Alum, Koen, Brea, Wild, Fish, and Pine) is accurate to 2 km and data obtained from the Canadian National Climate Data and Information Archive (for Fort, Tbay and Dryd) ranges in accuracy from 15 to 24 km.

**ESM Table 1** Site-level data with the number of sugar maple seedlings of northern, central, and southern origin that survived three years to harvest. Grayed-out site was not used in analyses. Pop., population.

|  |  | *#* Surviving Seedlings | | |  |
| --- | --- | --- | --- | --- | --- |
| Site name | MAT (°C) | Northern population | Central population | Southern population |  |
| ALUM | 16.1 | 29 | 33 | 38 |  |
| KOEN | 14.4 | 46 | 40 | 48 |  |
| BREA | 12.5 | 30 | 27 | 32 |  |
| WILD | 10.3 | 20 | 7 | 26 |  |
| FISH | 7.8 | 39 | 22 | 35 |  |
| PINE | 7.2 | 29 | 19 | 29 |  |
| PIKE | 3.9 | 23 | 0 | 5 |  |
| FORT | 2.9 | 39 | 4 | 21 |  |
| TBAY | 2.5 | 28 | 7 | 3 |  |
| DRYD | 2.1 | 19 | 7 | 9 |  |

**ESM Table 2** Summary of annual and summer climate data, calculated from 30-year climate normal (1971 – 2000), and soil characteristics typical of each site.

|  |  | Annual | |  | Summer | | |  |  | Soil | | |
| --- | --- | --- | --- | --- | --- | --- | --- | --- | --- | --- | --- | --- |
| Site | Lat.,  Long. | Temp. (°C) | PPT (mm) |  | Temp. (°C) | PPT (mm) | Moisture availability |  |  | Order, texture | pH |  |
| Jessieville, Arkansas  Alum Creek Experimental Forest  (Alum) | 34.8° N, 93.0° W | 16.1 | 1569 |  | 26.1 | 324 | 0.69 |  |  | ultisol,  loam | 4.1 |  |
| Jasper, Arkansas  Henry R. Koen Experimental Forest  (Koen) | 36.0° N, 93.2° W | 14.4 | 1196 |  | 24.8 | 268 | 0.62 |  |  | ultisol,  silt loam | 4.6 |  |
| Ashland, Missouri  Baskett Research & Education Area  (Brea) | 38.7° N, 92.2° W | 12.5 | 1023 |  | 24.4 | 293 | 0.69 |  |  | alfisol,  silt loam | 4.8 |  |
| Muscatine, Iowa  Wildcat Den State Park  (Wild) | 41.5° N, 90.9° W | 10.3 | 912 |  | 23.0 | 322 | 0.82 |  |  | alfisol,  silt loam | 6.5 |  |
| New Albin, Iowa  Fish Farm Mounds State Preserve  (Fish) | 43.5° N, 91.3° W | 7.8 | 853 |  | 21.1 | 335 | 0.99 |  |  | alfisol,  sandy loam | 6.8 |  |
| Marine-on-St. Croix, MN  Pine Needles tract  (Pine) | 45.2° N, 92.8° W | 7.2 | 815 |  | 21.2 | 346 | 1.00 |  |  | alfisol,  sandy loam | 6.8 |  |
| Neebing, Ontario  Private land  (Tbay) | 48.3° N, 89.4° W | 2.5 | 712 |  | 16.1 | 262 | 1.19 |  |  | brunisol,  sandy loam | 5.2 |  |
| Fort Frances, Ontario  Crown land  (Fort) | 48.8° N, 93.4° W | 2.9 | 721 |  | 17.4 | 305 | 1.20 |  |  | brunisol,  sandy loam | 4.6 |  |
| Dryden, Ontario  Crown land  (Dryd) | 49.9° N, 92.3° W | 2.1 | 701 |  | 17.4 | 289 | 1.13 |  |  | brunisol,  sandy loam | 4.5 |  |

*Notes*: Climate data were obtained from the PRISM Climate Group of Oregon State University (for the six sites in the United States) and the Canadian National Climate Data and Information Archive (for the three sites in Ontario, Canada). Summer moisture availability (PPT/PET) was calculated using equations from Thornthwaite (1948). Soil orders were determined from soil maps, while soil texture and pH were measured in the lab from soil samples collected at each site in 2011. Lat., latitude; Long., longitude; Temp., mean temperature; PPT, mean precipitation; PET, potential evapotranspiration.

**ESM Table 3** The effects of population (northern, central, and southern origin), clip treatment (clipped and unclipped understory), canopy cover type (closed canopy and canopy gaps), temperature (average summer temperature over the three years of the study at each of the transplant sites), and three-way interactions on sugar maple seedling root mass, accounting for differences in shoot mass (*R^2^* = 0.912, *N* = 683). In this mixed-effects model, both site and plot (nested within site) were entered as random effects to account for site-specific variation in seedling performance unrelated to temperature, and the random effects for plot were significant (*p* < 0.05). The model includes data from nine transplant sites spanning a latitudinal gradient with a 14 °C range in MAT. While the canopy cover type did have a significant effect on seedling root mass, differences between gaps and closed canopy plots in root mass fraction were tiny (both averaged 0.66), there were no significant interactions with temperature, and the AIC values were less negative for this model (-664.5) than those of the most conservative model where clip treatment and canopy cover type were omitted (-687.4). Hence we selected the simpler model to be shown as a table in the main text.

| Fixed effect | df | *F* | *p* | |
| --- | --- | --- | --- | --- |
| Population | 2, 563 | 107.1 | **<0.001** | |
| Clip treatment | 1, 581 | 3.1 | 0.080 | |
| Canopy cover type | 1, 584 | 7.3 | **0.007** | |
| Shoot mass (log) | 1, 642 | 903.8 | **<0.001** | |
| Temperature | 1, 9 | 0.2 | 0.632 | |
| Population x Clip | 2, 540 | 0.9 | 0.424 | |
| Population x Canopy cover | 2, 548 | 0.7 | 0.512 | |
| Population x Shoot mass | 2, 591 | 1.8 | 0.174 | |
| Population x Temperature | 2, 565 | 3.8 | **0.024** | |
| Clip x Canopy cover | 1, 292 | 0.0 | 0.992 | |
| Clip x Shoot mass | 1, 621 | 0.4 | 0.547 | |
| Clip x Temperature | 1, 413 | 1.0 | 0.327 | |
| Canopy cover x Shoot mass | 1, 614 | 4.4 | **0.037** | |
| Canopy cover x Temperature | 1, 436 | 0.6 | 0.442 | |
| Shoot mass x Temperature | 1, 642 | 1.0 | 0.323 | |
| Population x Clip x Canopy cover | 2, 504 | 0.2 | 0.806 | |
| Population x Clip x Shoot mass | 2, 533 | 0.1 | 0.941 | |
| Population x Clip x Temperature | 2, 554 | 0.1 | 0.891 | |
| Population x Canopy cover x Shoot mass | 2, 555 | 1.2 | 0.306 | |
| Population x Canopy cover x Temperature | 2, 563 | 0.4 | 0.678 | |
| Population x Shoot mass x Temperature | 2, 592 | 1.5 | 0.227 | |
| Clip x Canopy cover x Shoot mass | 1, 609 | 0.6 | 0.456 | |
| Clip x Canopy cover x Temperature | 1, 377 | 0.0 | 0.976 | |
| Clip x Shoot mass x Temperature | 1, 631 | 1.8 | 0.175 | |
| Canopy cover x Shoot mass x Temperature | 1, 621 | 2.5 | 0.112 | |

**ESM Table 4** Frequency and degree of cumulative summer soil moisture deficit over the past 115 years at a southern site (Lat. 39.0° N, Long. 90.9° W), a central site (Lat. 40.6 ° N, Long. 91.7° W), and a northern site (Lat. 45.9° N, Long. 93.6° W) whose climate matches that of the southern, central, and northern populations’ regions of origin. There is a higher frequency of summers with moisture deficit exceeding 200 mm at the southern than at the central or northern site. Moisture deficit is calculated by the difference between potential and actual evapotranspiration; moisture deficit is greater than zero in years where potential evapotranspiration is greater than precipitation. Climate data were obtained from the PRISM Climate Group of Oregon State University.

| Degree of Moisture Deficit | Summer Moisture Deficit Frequency  (# years), 1900 – 2014 | | | |
| --- | --- | --- | --- | --- |
|  | Southern site | | Central site | Northern site |
| 0 – 100 mm | 21 | | 33 | 77 |
| 101 – 200 mm | 50 | | 58 | 35 |
| 201 – 300 mm | 32 | | 19 | 3 |
| > 300 mm | 12 | | 5 | 0 |
